# Efficacy of botulinum toxin in benign essential Blepharospasm: Desirable & undesirable effects

**DOI:** 10.12669/pjms.296.3853

**Published:** 2013

**Authors:** M Shakaib Anwar, Humaira Zafar

**Affiliations:** 1 M Shakaib Anwar, Associate Professor of Ophthalmology, Rawal Institute of Health Sciences, Islamabad. Pakistan.; 2 Humaira Zafar, Assistant Professor of Microbiology, Al Nafees Medical College, Islamabad, Pakistan.

**Keywords:** Benign essential blepharospasm, botulinum toxin, facial dystonia, orbicularis oculi

## Abstract

***Objective: ***To study the efficacy, desirable and undesirable effects of locally injectable preparation of botulinum toxin in patients suffering from Benign Essential Blepharospasm (BEB).

***Methods: ***It was a prospective study carried out from October 2006 till November 2012 at a private set up, “Dr Shakaib’s Eye Clinic”, in Islamabad. Follow up of Seventeen patients of BEB has been done over six years period after injecting botulinum toxin. The patients had been explained about the study and informed consent was taken. After taking all the standard precautions for botulinum toxin injection, 5 to 7 (mean 6) sites for injecting 1.5 to 2.0 IU of the toxin were selected depending upon the severity and duration of the problem. These patients were requested to attend the clinic regularly, initially after three days and then weekly for two weeks, followed by every month for three months and then at three monthly intervals for up to six years.

***Results: ***The useful effects of the injection appeared in all the patients within 48 hours. There were minor side effects like irritation in the eyes and heaviness in the brow region. One (5.88 %) patient developed mild ptosis, which subsided over two weeks. Four (23.52 %) patients felt almost cured after three to four repetitions of injections. The useful effect of the injection lasted for about three months in all the patients.

***Conclusion: ***Botulinum toxin is a useful remedy for Benign Essential Blepharospasm (BEB), although the effects are short term and repeated applications are required which is quite costly for the patient.

## INTRODUCTION

Blepharospasm is a tonic spasm of the orbicularis oculi muscle which leads to intermittent or complete closure of the eyelids.^1^ Benign essential blepharospasm (BEB) is bilateral dystonia of orbicularis oculi muscles characterized by spasmodic involuntary eyelid closure in the absence of any other ocular or adnexal cause.^2^^,^^3^ It is not uncommon to see secondary blepharospasm, which is due to an ocular or adnexal disease, along with essential blepharospasm in the same patient.^4^ BEB is more common in women (2-3 times) as compared to men and more so in people over 50 years of age.^1^^,^^3^ Post menopausal women with thyroid dysfunction and those using phenothiazines are more prone to BEB.^4^ The common manifestations include photophobia, dry eyes, increased frequency of blinking which ultimately becomes spasmodic, leading to intermittent functional blindness. As the condition progresses, spasm may involve the midface over a variable period of time. This condition is referred to as Meige’s syndrome.^5^ The true pathologic basis of BEB is unknown,^5^^,^^6^ but abnormalities in basal ganglia and corticostriatopallidothalamic loop have been considered responsible and abnormal auditory brain stem response potentials have been noted in patients who have BEB.^7^^,^^8^

Presently the best modality available for the initial treatment of BEB is chemodenervation by botulinum toxin type A.^3^^,^^6^ Botulinum toxin Type A belongs to the group of seven antigenic – specific neurotoxins A,B,C1,D,E,F and G produced by Clostridium botulinum. It prevents acetylcholine release from the presynaptic terminals, thus blocks neuromuscular transmission at peripheral cholinergic nerve endings.^9^ The onset of effect takes about 24-72 hours although it may be delayed for 2-3 weeks.^6^^,^^10^^,^^11^ The plateau effect is achieved in about 3-5 days and usually lasts for three months.^9^^,^^10^ Generally, the quoted response rate to the injection is 95-98%.^11^ Antibodies formed to the toxin may lead to the failure of appropriate response in 2-5% cases. This occurs more when high doses are used at frequent intervals.^12^

**Fig.1:**
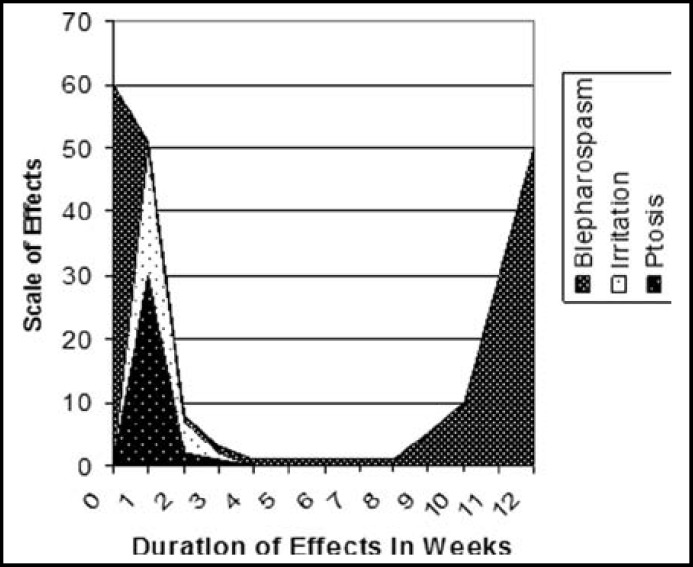
Scale and duration of desired & undesired effects of botulinum toxin

Usually procerus and corrugator muscles also require injection along with orbicularis muscle. One should be careful while injecting in the upper lid for fear of migration of toxin to the levator muscle which may lead to temporary ptosis. The recommended initial dose for type A toxin is 1.25-5 units per injection site. This dose may be increased if the response is not sufficient. The usual sites for injection are depicted in the Fig.1. Recovery of muscular function occurs in about three months time by axonal sprouting and formation of new neuromuscular junctions.

It is recommended that the toxin injection should not be repeated before three months and the dose should not exceed 200 units in 30 days period. In such cases toxin type F can be effective. Upper lid myectomy is a good alternative in such cases.^13^ Alternatively dose and injection sites can be increased or changed or oral medication can be given along with injections.^14^ A different ‘no-protein’ formula of botulinum toxin has been developed to avoid antibodies formation. The dose of this formula is 3-4 times that of the usual one.^15^ One study has revealed that a dose of 80 units per eye (about 10 units per site) has better sustained effect with this new form.^16^

 Adverse reactions to the botulinum toxin include ptosis, keratitis, epiphora, diplopia and ocular irritation. These are transient and usually do not last more than three weeks. Other less common side effects include transient increase in intraocular pressure, flu-like syndrome and secondary biliary colic.^10^

## METHODS

This study has been carried out over about six years on 17 patients of BEB, out of which 2 (11.76%) were males and 15(88.23%) were females. All of them aged between 45 and 65 years (mean age 55) having well established BEB with the history of complaints from six months to two years. One (5.88%) patient had additional slight involvement of lower face muscle and 2 (11.76%) had spasm in the frontalis muscle as well.

The follow up period ranged between 6 months to 6 years (mean 39 months), the period being different for every patient. Some patients had also been photographed after taking consent and the pictures have been masked to hide the identity.

The inclusion criteria for the patients included in this study were: (i) idiopathic (essential) blepharospasm (ii) no neurologic or psychiatric disease and (iii) no history of any surgical intervention for BEB. The non-willing patients were excluded from the study.

The patients had been explained about the study and informed consent was taken. The patients were explained about the effects, possible side effects and the expected duration of the useful effects of the injection. These patients were requested to attend the clinic regularly, initially after three days and then weekly for two weeks and then every month for three months and later on every three months for repeating the injections.

The injection was prepared by adding 2 ml of preservative free 0.9% saline solution to 100 units of purified dry botulinum toxin supplied in a sealed glass vial with a negative pressure inside. This would give 5 units of toxin per 10^th^ division of 27 G, 1 ml insulin syringe. Precautions were taken to maintain the cold chain of supply (around 4 degrees Celsius).

All the patients received subcutaneous injections at 5-8 sites under aseptic conditions and taking standard precautions for injection procedure. The toxin was injected initially at five selected sites in the periocular region and the starting dose in all the patients was 2 units per injection site. Later on the dose and the sites had to be increased to 6 units and 8 sites respectively according to the individual response of the patients.

## RESULTS

**Fig.2:**
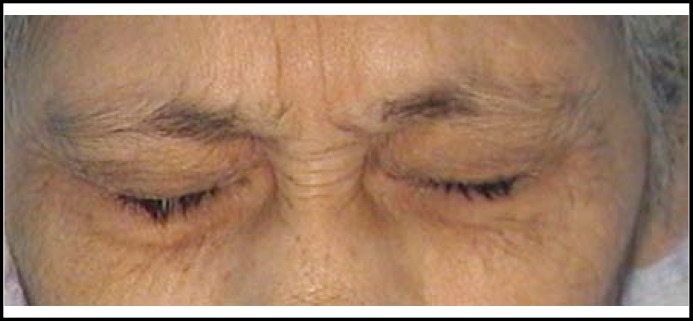
Photograph of the patient before injection

The data was analyzed by SPSS version 16. Frequency of specific case was assessed in terms of percentages for qualitative variable and statistical inference. The injection showed its effects in all the patients within first 48 hours. One (5.88%) patient developed ptosis on the third day which settled over two weeks. Nine (52.94%) patients experienced optimal results i.e. elimination of spasm and 8 (47.05%) patients experienced the reduction of spasm to tolerable level (Table-I, Fig.2). Two (11.76%) patients with the frontalis involvement experienced relief in spasm of that region but 1 (5.88%) patient with involvement of lower face experienced optimal results for blepharospasm but the lower face muscles did not respond well to the treatment. All (100%) the patients experienced mild irritation in the eyes and a mild sense of heaviness over the brow region. These undesirable effects lasted, on an average, for two to three weeks.

**Table-I:** Effects of Botulin Toxin Injection (Observed within 48 hours) (n=17).

*Effects*	*Number of Patientsn (%)*
Complete relief of spasm	9 (52.94%)
Partial Relief to tolerable level	8 (47.05%)
Mild to moderate Irritation in the Eyes	17 (100%)
Ptosis	1 (5.88%)

**Fig.3:**
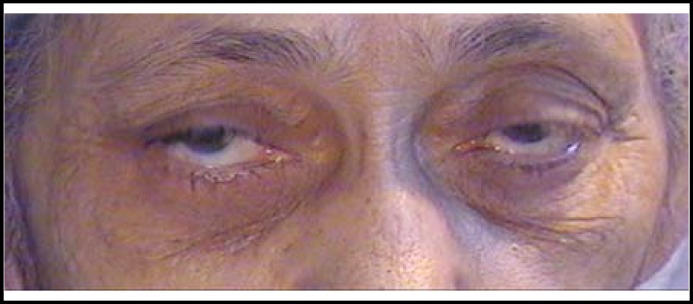
Photograph of the patient one week after injection

**Fig.4:**
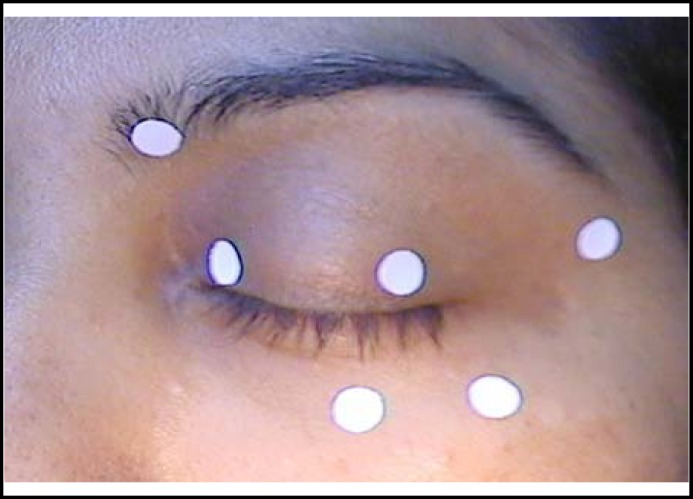
Usual sites of injection for botulinum toxin

The useful effect of relief from the BEB continued till 10 to 12 weeks. Four (23.52%) patients felt almost cured and the spasm dropped down to acceptable level after three to five repetitions of injection and stopped further treatment. Eight (47.05%) received the treatment regularly at three months interval for two to three years and then stopped further treatment. They experienced that the symptoms had reduced to tolerable level. Four (23.52%) patients had been irregular in treatment but continued treatment for two to three years with satisfactory results. One female patient was still getting regular treatment and had received 21 treatments as she entered into 6^th^ year of follow up. After second or third treatment the dose was to be increased to 4 units per injection in all (100%) cases. In the longer treatment group (two to more than five years) the number of injection sites were to be increased to seven or even eight. This depended on the response of the previous treatment. With every treatment session, the patients response was variable although with in satisfactory limits. The muscle mass under the skin appeared to be reduced in the longer treatment group especially the one who received the maximum injections.

## DISCUSSION

Benign essential blepharospasm is usually misdiagnosed by the general practitioners and the patients are treated with partially effective or ineffective remedies like anxiolytic or sedative agents. A very small percentage of cases is able to get the advice of the desired physician or ophthalmologist who can manage the disease properly.

Botulinum toxin A has been established as the first line treatment of choice for BEB. It has been found safe and effective for long term treatment of BEB if injection intervals and dosages are chosen carefully.^17^ In our study we have tried to observe its efficacy in our patients and to critically analyze the duration of its useful effect, undesirable side effects and any changes required in the dose or injection sites over the passage of time. Generally, we found it very effective in control of BEB, although the treatment had to be repeated every three months. The side effects like dryness and irritation were short term and could be controlled easily.. When repeatedly injected over long time, we had to increase the dose (in units) per injection site. This was probably due to the loss of efficacy of the toxin due to antibodies formation.^12^ The length of symptom free period with every application remained almost the same over long term treatment. Similar results have been shown in studies carried out by Czyz, Burns and Ainsworth.^18^^,^^19^ Due to the long symptom free periods, our patients experienced improved quality of life, both socially and physically. Our experience in this aspect was not different from studies carried out at other centers where Blepharospasm Disability Index (BSDI) scoring was done.^20^ One of our patients felt relief in chronic frontal headache after the injection. The same was also noted by Vogt et al, Alajbegovicetal and Harrison et al in their study model.^21^^-^^24^

Ptosis and other side effects like keratitis, epiphora and raised intraocular pressure were less common. In one study the occurrence of ptosis has been reported in 22% of the cases^10^, while in our study only one (5.88%) patient experienced transient ptosis. This might be due to overdose or slightly different technique. We have seen that staying away from the centre of the eye lid reduces the risk of ptosis. Price and Farish have suggested that less ocular side effects occur when the injections are applied away from the eyelid margin.^25^ One study has revealed improved tear film stability and increased tear meniscus height (TMH) in BEB patients treated with botulinum toxin injection, thereby improving the symptoms of dryness associated with the disease.^26^

 A national study carried out for facial dystonias, included four patients of BEB and found botulinum toxin effective in its treatment. The side effects experienced by the patient of this study were similar but less in frequency to those experienced by our patients. ^27^

Our current study results regarding the efficacy and side effects of botulinum toxin for BEB are consistent with studies carried out at other centers.^9^^,^^28^

The limitations of our study included relatively small number of cases due to infrequent referrals, low patient compliance and very little local data available on the subject for comparison.

## CONCLUSION

Botulinum toxin is the treatment of choice as the first line therapy for BEB. The only drawback is that the effect wears off in about 12 weeks and the injections are to be repeated every three months. This demands patients’ motivation and affordability.

## Notes

***Funding Source:*** Self-funded (all the kits and reagents were purchased on self- funded grounds).
